# A randomised feasibility study of serial magnetic resonance imaging to reduce treatment times in Charcot neuroarthropathy in people with diabetes (CADOM): a protocol

**DOI:** 10.1186/s40814-020-00611-3

**Published:** 2020-06-16

**Authors:** Catherine Gooday, Frances Game, Jim Woodburn, Fiona Poland, Erika Sims, Ketan Dhatariya, Lee Shepstone, Wendy Hardeman

**Affiliations:** 1 School of Health Sciences, University of East Anglia, Norwich, NR4 7TJ UK; 2 Elsie Bertram Diabetes Centre, Norfolk & Norwich University Hospitals NHS Foundation Trust, Norwich, NR4 7UY UK; 3 Department of Diabetes and Endocrinology, University Hospitals of Derby and Burton NHS Foundation Trust, Derby, DE22 3NE UK; 4 School of Health and Life Sciences, Glasgow Caledonian University, Glasgow, G4 0BA UK; 5 Norwich Clinical Trials Unit, Norwich Medical School, University of East Anglia, Norwich, NR4 7TJ UK; 6 Norwich Medical School, University of East Anglia, Norwich, NR4 7TJ UK

**Keywords:** Charcot neuroarthropathy, Diabetes, MRI, Temperature monitoring, X-ray, Patient experience, Feasibility study

## Abstract

**Background:**

Charcot neuroarthropathy is a complication of peripheral neuropathy associated with diabetes which most frequently affects the lower limb. It can cause fractures and dislocations within the foot, which may progress to deformity and ulceration. Recommended treatment is immobilisation and offloading, with a below knee non-removable cast or boot. Duration of treatment varies from six months to more than 1 year. Small observational studies suggest that repeated assessment with magnetic resonance imaging improves decision-making about when to stop treatment, but this has not been tested in clinical trials. This study aims to explore the feasibility of using serial magnetic resonance imaging without contrast in the monitoring of Charcot neuroarthropathy to reduce duration of immobilisation of the foot. A nested qualitative study aims to explore participants’ lived experience of Charcot neuroarthropathy and of taking part in the feasibility study.

**Methods:**

We will undertake a two-arm, open study and randomise 60 people with a suspected or confirmed diagnosis of Charcot neuroarthropathy from five NHS, secondary care multidisciplinary Diabetic Foot Clinics across England. Participants will be randomised 1:1 to receive magnetic resonance imaging at baseline and remission up to 12 months, with repeated foot temperature measurements and X-rays (standard care plus), or standard care plus with additional three-monthly magnetic resonance imaging until remission up to 12 months (intervention). Time to confirmed remission of Charcot neuroarthropathy with off-loading treatment (days) and its variance will be used to inform sample size in a full-scale trial. We will look for opportunities to improve the protocols for monitoring techniques and the clinical, patient-centred and health economic measures used in a future study. For the nested qualitative study, we will invite a purposive sample of 10–14 people able to offer maximally varying experiences from the feasibility study to take part in semi-structured interviews to be analysed using thematic analysis.

**Discussion:**

The study will inform the decision whether to proceed to a full-scale trial. It will also allow deeper understanding of the lived experience of Charcot neuroarthropathy, and factors that contribute to engagement in management and contribute to the development of more effective patient-centred strategies.

**Trial registration:**

ISRCTN, ISRCTN74101606. Registered on 6 November 2017.

## Background

Charcot neuroarthropathy (CN) is a complication of peripheral neuropathy associated with diabetes which most frequently affects the lower limb. It can cause fractures and dislocations within the foot, which may progress to deformity and ulceration. The symptoms include redness, warmth and swelling in the foot and/or leg. This inflammation can lead to fractures in the bones and can damage joints, affecting the shape and function of the foot. It was first described 140 years ago [1]; however, it remains a poorly understood and frequently overlooked complication of diabetes [2].

Population-based studies have estimated a life time cumulative incidence for CN of 0.4 to 1.3% in people with diabetes, rising to 13% in people at high risk who attend diabetic foot speciality clinics [3]. In 2018, a regional survey of 205,033 people with diabetes in the East Midlands, UK, reported a point prevalence of 0.04% [4]. CN is associated with increased length of stay and use of medical resources [5].

The aim of treatment is to stop the inflammatory process, relieve pain and maintain foot architecture and so reduce the risk of future ulceration and amputation [6]. The current international consensus is that the foot should be immobilised in a below knee non-removable cast or boot, with weekly or fortnightly review by healthcare professionals working in specialist multidisciplinary diabetic foot clinics [7]. The immobilisation minimises the potential for any further damage to the foot structure. Immobilisation is continued until remission, defined as the absence of clinical signs of inflammation, measured using skin surface infra-red thermography and X-rays showing signs of bone healing and union [8].

The evidence base for the treatment of CN is weak. It is based on studies from a few centres which used retrospective designs and case note review methods using small sample sizes, typically in the range of 9–55 participants [3, 9–13]. Many studies failed to standardise monitoring, treatment and outcomes, which makes direct comparison between studies difficult.

Studies from the UK have shown a median time to remission of 9–12 months [9, 13, 14]. However, US studies report considerably shorter time to remission of 3–5 months [3, 10–12]. Studies from Brazil and Germany show remission times of 3–12 months and 3–6 months, respectively [15, 16]. Shorter treatment times could be related to reported differences in the relapse rates for CN, between 12 and 33% [13, 17–19], but without clear and consistent definitions for remission and relapse, this is unknown. There is also variation in the reported annual major amputation rates in people with CN from two different case series from hospitals in the USA—2.7% and 6.6% [20, 21].

The reasons for the variation are not understood but could include people’s characteristics at the start of the treatment, different techniques for monitoring CN, different protocols for the same monitoring techniques, variations in approach to off-loading and variability in study design. These could either underestimate or overestimate treatment duration.

Temperature difference between the feet is one of the most frequently used methods to monitor CN. It is recommended in the 2015 National Institute for Health and Care Excellence guidance on diabetic foot problems [22]. The most recent systematic review [8] published in 2013 recommends that immobilisation is continued until the temperature difference between the feet is less than 1–2 °C, and no further radiological changes on imaging have occurred. However, this recommendation is only based on level IV evidence, i.e. case series [8]. There is variability in the protocols used to measure the temperature difference between the feet. The most detailed protocol for measuring temperature discrepancy requires a 15-min acclimatisation period, controlled ambient air temperature and readings collected from nine different places on each foot [23]. In addition, plain X-rays demonstrate damage to the bone and joints rather than disease activity (inflammation).

Studies show inconsistency in the methods for monitoring and monitoring devices used [13, 17–19, 23–25]. These factors may overestimate or underestimate the degree of inflammation, so treatment may be discontinued too early or continued for longer than necessary. The presence of simultaneous bilateral foot disease or the absence of a contralateral limb through prior amputation invalidates the use of temperature measurement as a tool for identifying disease remission.

The National Institute for Health and Care Excellence recommends the use of MRI in determining a diagnosis of CN in the early stages of disease when no signs are evident on plain radiology [22]. However, serial MRI is not widely used in routine clinical practice as a tool to monitor for signs of disease remission in CN [26]. One prospective study using MRI with contrast reported that mean healing times were associated with contrast uptake assessed at baseline [27]. A further two retrospective studies looked at bone marrow oedema. One study reported decreasing bone marrow oedema in 69% of follow-up images [28], and the second study found a significant positive correlation between intensity of bone marrow oedema on MRI and clinical measures [29]. This emerging evidence suggests that MRI may be useful for the surveillance of active CN. The findings from MRIs could be adopted as the criterion standard for establishing disease activity and remission.

The use of MRI in monitoring CN therefore needs to be formally evaluated in a trial [30]. However, the evidence to support a full randomised controlled trial is presently insufficient. We will conduct a randomised feasibility study to understand the proportion of people who meet the eligibility criteria, the number of eligible participants recruited, the number of participants who receive an alternative diagnosis and the proportion of participants who withdraw. Time to MRI confirmed remission of CN with off-loading treatment (in days), and its variance will be used to inform sample size in a main trial. We will look for opportunities to improve the protocols for monitoring techniques in a future trial. We will examine the feasibility of a range of clinical, patient centred, and health economic measures. We are using a randomised controlled trial as it is considered the gold standard for evaluating efficacy in clinical research [31].

As part of the feasibility study, we will carry out a qualitative study to further the understanding of people’s experiences of living with CN and the factors that contribute to people’s engagement in their treatment. Previous qualitative studies have demonstrated the importance of people’s perspectives in order to promote engagement in the prevention and management of diabetic foot ulcerations [32–34]. What may be people’s views and experiences of CN is an under-researched area [35]. In the UK treatment times for CN are between 9–12 months [14], which is longer than those for foot ulceration, where treatment times are no more than 12 weeks for half of the people [36]. This means that evidence on people’s experiences of foot ulceration may not transfer to CN.

In summary, there is a lack of evidence to support the use of monitoring techniques in CN. Healthcare professionals rely on methods and devices which do not accurately reflect disease progression, and decision-making about discontinuing or prolonging immobilisation is challenging. A lack of understanding of people’s experiences of living with CN means their needs and wishes may be neglected with current treatments, and are not being considered when developing new treatment strategies and pathways.

## Aim and objectives

This study aims to explore the feasibility of using serial MRI without contrast in the monitoring of CN to reduce duration of immobilisation of the foot, in order to decide whether a large-scale trial is warranted. We will assess eligibility, recruitment, retention and withdrawal rates. Time to MRI confirmed remission of CN with off-loading treatment (days), and its variance will be used to inform sample size in a main trial. We will also examine the feasibility of collecting clinical, patient-centred and health economic measures. The nested qualitative study aims to explore the dimensions of lived experience of CN and the participants’ experiences of taking part in the feasibility study.

## Methods

### *Study design* (Fig. 1)

**Fig. 1:**
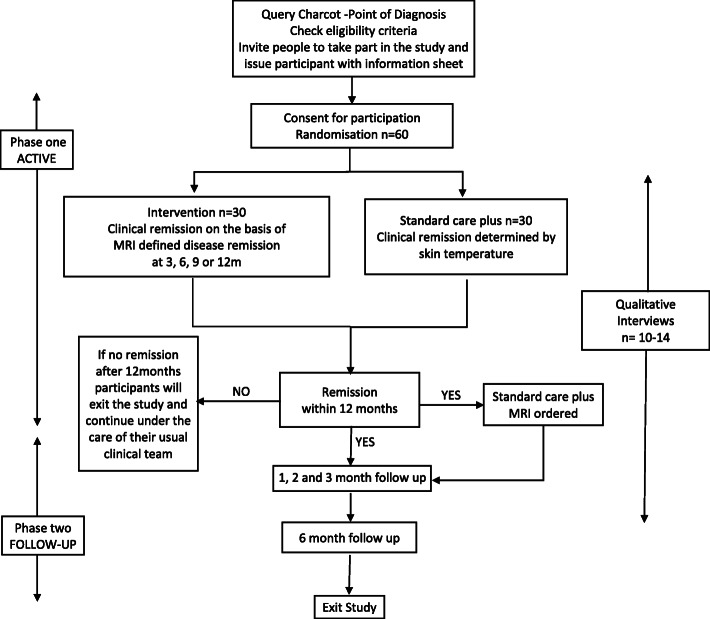
Patient flow diagram

This is a two-arm, open, randomised controlled trial investigating the feasibility of using serial MRI to monitor CN. The study will last for a maximum of 3½ years. The study is divided into two phases: phase one, the active phase, will last until the CN is in remission or a maximum of 12 months, and phase two, the follow-up phase, will last for six months after remission (Fig. 1). The maximum time a participant will be in the trial is 18 months.

The decision to use an open label design was pragmatic: the MRIs will be reported by radiologists and interpreted by the healthcare professionals working in multidisciplinary specialist diabetic foot clinics. As the reporting of MRIs relies on comparison to previous images, this will indicate the trial arm the participant has been randomised to.

The trial has been reviewed and approved by East Midlands-Derby Research Ethics Committee, April 10, 2017, ref 17/EM/0288.

### Setting

The setting will be multidisciplinary specialist diabetic foot services at five NHS Hospital Trusts in England.

### Randomisation

A randomisation scheme has been generated by the trial statistician. Allocation will be stratified by centre. Participants will be randomised using a web-based randomisation process on a 1:1 basis to (a) immobilisation discontinued on the basis of clinical remission and determined by skin temperature measurement, which triggers an MRI (standard care plus) or (b) standard care plus and additionally the serial use of MRI at 3, 6, 9 and 12 months to identify disease remission and thus discontinuation of immobilisation (intervention).

### Sample size

As this is a feasibility study, a power calculation is not required. An allowance has been made for up to 10–15% of participants to be withdrawn from the study due to an alternative diagnosis. The sample size will be 60 people with 30 participants per arm, based on recommended sample sizes between 24 and 50 for a feasibility study [37, 38]. We will invite a purposive subsample of 10–14 participants from the feasibility study to take part in the qualitative study.

### Participants—inclusion and exclusion criteria

Participants will be people with diabetes as defined by the World Health Organisation [39] and a suspected or confirmed diagnosis of CN who are attending NHS multidisciplinary specialist diabetic foot services. They will be identified, recruited and consented by the healthcare professionals working in the foot clinics, and these will include podiatrists, nurses and doctors. The full inclusion and exclusion criteria are shown in Table 1. The main exclusion criteria were selected because (1) they are contra-indications to having an MRI scan, (2) bilateral disease prevents temperature comparison with the contra-lateral limb, and (3) co-morbidities may alter people’s inflammatory response. A confirmed diagnosis of CN can take several weeks, so participants will be recruited as early as possible to accurately collect length of time in below knee non-removable cast or boot. If the clinical team decides on an alternative diagnosis during the trial, then the participant will exit the study. We anticipate that alternative diagnosis will include infection, gout, arthritis, soft tissue injuries or deep vein thrombosis. Follow-up care will be provided by the appropriate clinical team.

**Table 1:** Inclusion and exclusion criteria

Inclusion criteria	Exclusion criteria
Participants who are willing and have capacity to give informed consent.	People who have received a transplant and others receiving immunosuppressant therapy or using long-term oral glucocorticoids other than in the routine management of glucocorticoid deficiency. Participants on a low dose of oral glucocorticoids (< 10 mg for ≤ 7 days) are eligible to participate in the study.
People with diabetes as diagnosed by the WHO criteria http://www.who.int/diabetes/publications/diagnosis_diabetes2011/en/	Participation in another intervention study on active CN.
Age 18 years or over.	Contra-indication for MRI.
New or suspected diagnosis of acute CN (no previous incidence of acute CN within the last 6 months on the same foot) treated with off-loading.	Treatment for previous suspected CN on the same foot in the last 6 months.
Understand written and verbal instructions in English.	Suspected or confirmed bilateral active CN at presentation.
	Active osteomyelitis at randomisation.
	Previous contralateral major amputation.
	Inability to have an MRI scan.
	People receiving palliative care.

For the qualitative study, we have identified five participant characteristics which will purposively inform the sampling framework and will seek to maximise variation in sex, age, history of previous foot complications, duration of treatment for the current episode of CN and employment status. In addition to these factors, we will also ensure that participants equally represent both study arms.

### Outcomes

We will measure a range of feasibility, clinical efficacy and patient centred outcomes (Table 2). We will record time to MRI confirmed remission of CN with off-loading treatment (days), and its variance will be used to inform the sample size for a full-scale trial.

**Table 2:** Feasibility, clinical efficacy and patient centred outcomes

Feasibility outcomes	Clinical efficacy outcomes (collected at all study visit)	Patient centred outcomes (collected at baseline, 3 monthly until remission, then at 1 and 6 months post remission)
The proportion of patients who meet the eligibility criteria	Number of new ulcerations on the index or contralateral foot	Health-related quality of life measured: Short Form 12 questionnaire (SF-12) [40] EuroQol-5D-5 L questionnaire (EQ-5D-5 L) [41]
The number of eligible patients recruited	Number of new infections on the index or contralateral foot	Hospital Anxiety and Depression Scale (HADS) [42]
The number of participants in which an alternative diagnosis is made during the active phase of the trial	Number of minor and major amputations on the index or contralateral foot at the end of the follow-up phase of the study	Pain as assessed by Visual Analogue Scale (VAS)
The proportion of patients that withdraw or are lost to follow-up. The term ‘withdrawal’ encompasses two potential scenarios: withdrawal due to loss of consent or withdrawal due to deaths	Number and severity of falls (Hopkins Fall Grading System) [43]	Patient diary
Statistical parameters of the key outcome measures, duration in off-loading to inform a sample size calculation for a definitive trial	The number of participants in each arm requiring further intervention for CN (e.g. further immobilisation) within 6 months of remission	
Ability to collect quality of life and resource use data		

For participants in the standard care plus arm, remission is defined as a temperature difference of ≤ 2 ^°^C which is maintained or improves on two separate consecutive occasions for a period of at least four weeks [8] or at the discretion of the clinical team when temperature difference is not valid; for example in the presence of bilateral foot disease. In the standard care plus arm, this will then trigger an MRI. In the intervention arm remission is defined as an absence of subchondral bone marrow oedema on MRI, as reported by a radiologist and the absence of clinical signs and symptoms of CN. The clinical team will interpret the results of the MRI report to determine remission.

The final visit will be six months after remission. During these six months, we will continue to monitor the foot using the standardised assessment of foot temperature for any clinical signs that the CN has relapsed. We have defined relapse as a temperature difference of > 2 °C compared to the contralateral foot maintained for two or more occasions or further changes on imaging. The final decision as to whether the CN has relapsed will be at the discretion of the clinical team.

We will explore the feasibility of collecting resource use and quality of life data, to inform the design of the health economics component of a future definitive trial. Data on all primary care and secondary care visits and admissions to hospital will be collected. Time off work and levels of informal care will also be assessed. We will use the qualitative interviews to gain a deeper, more detailed and rounded contextualised understanding of participants’ lived experience of CN and of taking part in this study.

### Planned interventions

**Fig. 2:**
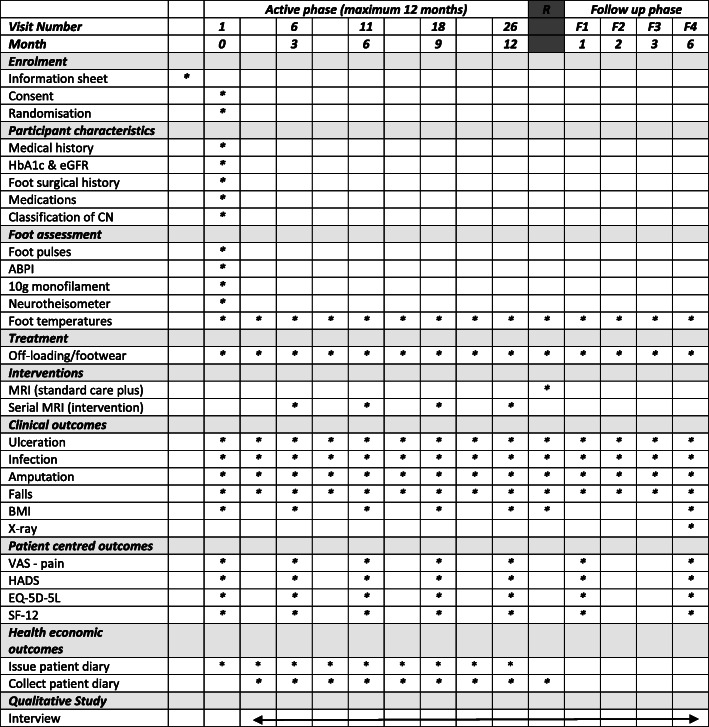
Schedule of enrolment, interventions and assessments. Active phase - while the CN is active participants will attend every 14 days, up to a maximum of 26 visits. Follow up phase – once CN is in remission participants will transfer into the follow-up phase of the study for six months. Classification of CN – accordingly to the Sanders and Frykberg and the modified Eichenholtz classification tools

*Standard care plus* participants will receive standard care for the assessment and management of CN and any other foot problems; alongside this, we will collect study measures (Fig. 2). If participants have not had a recent diagnostic X-ray or MRI (within the last 3 weeks, prior to randomisation), this will be requested. In this study, we have standardised the assessment of foot temperature to monitor CN by using the same device, the Thermofocus 01500A3®. Every 14 days, the temperature of both feet will be recorded at intervals of 5 min, starting at the removal of the off-loading device and up to 15 min. The sites where the temperature will be measured are based on the classification tool developed by Sanders and Frykberg [44]. We will classify the stage using the modified [45] Eichenholtz classification tool [46] and location of the CN [44] at baseline using anterior/posterior, oblique and lateral weight bearing X-rays.

*Intervention* in addition to standard care plus, participants in the intervention arm will receive serial MRIs at 3, 6, 9 and 12 months. Intervention participants will not undergo further MRIs once remission has been diagnosed, i.e. if remission is diagnosed at 6 months, the MRIs at 9 and 12 months will not occur.

### Study procedures (Fig. 2)

The schedule of enrolment, interventions and assessments is shown in Fig. 2. After giving written informed consent (see Additional file 2), participants will attend for visits every 14 days until remission. All visits will take place in multidisciplinary foot clinics. Wherever possible, study measurements and trial interventions will coincide with the participant’s existing clinic appointments. This will reduce study burden which is likely to help increase recruitment and retention rates. The study protocol (v1.3, dated 22 July 2019) is based on the Standard Protocol Items: Recommendations for Interventional Trials (SPIRIT) 2013 Statement for protocols of clinical trials (see Additional file 1).

Prior to participating in the interviews about the lived experience of CN, participants will receive a further patient information sheet explaining the purpose of the interview and will be asked to complete another consent form (see Additional file 3). All the qualitative interviews will be carried out by the first author (CG), using a semi-structured approach. The topic guide will include a number of probes designed to prompt the participant to increase the level of detail and depth of the information provided from the participants’ own viewpoint. Interviews will last approximately 30–40 min in a place of the participant’s choosing. The interviews will be audiotaped (with the participant’s permission) and transcribed in full to capture language and their own expressions.

### Analyses

#### Quantitative analysis

The feasibility measures including eligibility, recruitment, retention and withdrawals will be reported as point estimates with 95% confidence intervals. There is no intention to conduct any formal comparative analyses for these measures, though levels of missing data will be explored with respect to certain baseline characteristics, e.g. age and measures of disease severity. Variability in outcomes (e.g. standard deviation) will be estimated with 95% confidence intervals to inform the sample size calculations for a full-scale trial. Any between-group efficacy analyses will only be exploratory. There are no plans for any interim analyses.

We will assess progression of foot deformity by comparing X-rays at baseline, remission and six months post remission. We will measure the change in the calcaneal inclination, talar declination and talo-first metatarsal angle between the X-rays. People who have undergone previous minor amputation and/or previous orthopaedic surgical fixation of the foot which alters or removes the anatomical landmarks of the foot will be excluded from this analysis due to the absence of bony landmarks.

The main purpose of the economic analysis is to inform how the data on costs and effects would be collected within a definitive study. Thus, we will estimate completion rates and seek to identify big cost drivers, in order to inform this decision. A preliminary cost-effectiveness analysis will also be performed, although the findings will be treated with caution. As such, we will estimate the mean incremental cost and mean QALY gain associated with the intervention compared to standard care plus.

#### Qualitative analysis

The qualitative interviews will be analysed using inductive thematic analysis using the six-step model [47]. The first author (CG) will read all the transcribed interviews to record emerging ideas. The interviews will then be subjected to line-by-line coding using the NVivo data management package. The coding framework will be refined by a second researcher, who will cross-check it against a small sample of transcripts. A modified framework approach will be used to organise the analysis. The coded data will be subjected to a thematic analysis, identifying key categories and themes from the data, ensuring that all participants’ responses are adequately captured and their meaning authentically interpreted. This approach will provide rich descriptions of the data representing accounts of the diverse and personal experiences of people who have taken part in the study and been treated for acute CN.

#### Data management and quality assurance

We will set up a Trial Management Group to assist with co-ordination and strategic management of the feasibility study. An initial on-site initiation visit will be completed by CG prior to the sites opening. The primary method of data collection by the research teams will be direct online entry of data onto a purpose-designed secure password-protected electronic case record form. The database complies with data protection requirements [48] on confidentiality and anonymity. Quality management and monitoring procedures have been discussed and agreed with the sponsor. Central monitoring has been considered appropriate for this study with the option to escalate findings and conduct ‘for-cause’ on-site triggered monitoring visit if indicated. We will review completed consent forms and selected data points for quality assurance at each site within a week after randomisation of the first participant. Subsequent monitoring will be completed at six monthly intervals to coincide with the Trial Management Group meetings and at the end of data collection.

#### Safety reporting

Safety monitoring and reporting of adverse events has been discussed and agreed with the sponsor. The study has been assessed as low risk; therefore, there will not be a data monitoring committee. The intervention consists of increased frequency of MRI scans without contrast, so a pragmatic approach to safety reporting will be used. MRI scans will be performed in NHS hospitals under routine clinical protocols. Adverse events resulting from MRI scans will be reported by the research teams in line with the Hospital Trust’s clinical incident reporting policy. A copy of the anonymised incident form will be forwarded to the Chief Investigator (CG) and reviewed by the Trial Management Group. All other anticipated events, e.g. ulceration, infection, amputation, pain, falls and death will be recorded as secondary outcomes.

## Discussion

CN is a poorly understood and under researched complication of diabetes, associated with increased morbidity and mortality compared to people with diabetes without peripheral neuropathy. Evidence is lacking about the factors that influence the unexplained variation in treatment times, relapse rates and complications such as ulceration and amputation. We have also identified a lack of evidence to support the efficacy of current monitoring techniques in CN. There is evidence from small studies that MRI may be superior to current methods of monitoring for remission in CN, but this has not been formally evaluated using robust designs. The results of this feasibility study will inform the decision about progressing to a full-sized pragmatic randomised controlled trial: the number of sites required, trial design, the frequency of MRI monitoring and the choice of process and outcome measures. The embedded qualitative study will provide contextual and meaningful insight into people’s experiences of living with CN and what factors they see as contributing to their engagement with the prescribed treatment. Secondly, the qualitative study will advance our understanding of how the condition impacts on participants’ quality of life and may contribute to future work on patient reported outcomes measures in this area [49]. Finally, the findings from the qualitative study will provide additional insights into aspects of the trial design and processes that could be improved, in terms of engagement of, and acceptability to participants, based on the participants’ experience of involvement in the feasibility study. These aspects could include feedback on the frequency of trial visits, the length of the active and follow-up phases of the trial and the choice and frequency of completing validated questionnaires. The results of this study will be disseminated to researchers, clinicians, people with diabetes and relevant stakeholders through presentations, publications and social media press releases.

### Trial status

The CADOM trial originally opened for recruitment in December 2017 and is currently recruiting participants. Recruitment will continue until the end of November 2019.

## Supplementary information

**Additional file 1.** SPIRIT Checklist.

**Additional file 2.** Informed consent form—feasibility trial.

**Additional file 3.** Informed consent form—qualitative interviews.

## Data Availability

The datasets generated and/or analysed during the current trial will be available from the corresponding author on reasonable request, provided appropriate credit is attributed to the original authors and the data source.

## Abbreviations

ABPI: Ankle brachial pressure index
BMI: Body mass index
CN: Charcot neuroarthropathy
eGFR: Estimated Glomerular filtration rate, ml/min
EQ-5D-5 L: Euroqol 5D
F: Follow-up visit
HADS: Hospital Anxiety and Depression Scale
HbA1c: Glycated haemoglobin (A1c), mmol/mol
MRI: Magnetic resonance imaging
NHS: National Health Service
R: Remission
SF-12: Medical Outcomes Short-Form Health Questionnaire
VAS: Visual Analogue Scale
