# Supplementary material for: A randomised feasibility study of serial magnetic resonance imaging to reduce treatment times in Charcot neuroarthropathy in people with diabetes (CADOM): a protocol
Source: Pilot Feasibility Stud. 2020 Jun 16;6:85. doi: 10.1186/s40814-020-00611-3 (PMC7296621; doi:10.1186/s40814-020-00611-3)
Supplement: Supplementary file 2 — Additional file 2. Informed consent form—feasibility trial. [file 40814_2020_611_MOESM2_ESM.docx]

*Insert local header*

**A study to assess the use of serial MRI to reduce treatment times in Charcot in people with diabetes.**

**(Short title: CADOM)**

**Charcot neuroArthropathy Diagnostic Outcome Measures**

**Patient Consent Form**

Principal Investigator:……………………………………………………..

Patient Study ID: …..……………….. Initials: ………………

Please initial each box

1. I confirm that I have read and understand the information sheet

Version 1.2 10^th^ January 2019 for the above study. I have had the

opportunity to ask questions and been given satisfactory answers.

1. I have been given a full explanation of the purpose of the study and

what I will be expected to do.

1. I understand that my medical notes and data collected during the study

YES

NO

may be looked at by individuals from the Clinical Trials Unit at the

University of East Anglia, from regulatory authorities or from the NHS

Trust, where it is relevant to my taking part in this research I give

permission for these individuals to have access to my records.

1. I understand that my participation is voluntary and that I am free to

withdraw at any time without my medical care or legal rights being

affected

1. I consent to the storage including electronic, of personal information for

the purposes of this study. I understand that any information that could

identify me will be kept strictly confidential and that no personal

information will be included in the study report or other publication.

1. I understand that even if I withdraw from the above study, the data

collected from me up to that point will be used in analysing the results

of the study.

1. In the event that the MRI or X-ray shows a previous unknown condition that might need further medical or surgical intervention I agree to the research team referring me on as necessary and informing my GP.
2. I understand that information held by the NHS and records maintained

by the NHS Information Centre may be used to keep in touch with me and

my health status. I give my permission to register my identifiable details

with the NHS Information Centre.

NO

YES

1. I agree to being contacted by the research team when the Charcot

has settled, to ask if I would consider taking part in an interview.

The interview would involve discussing the experience of being

diagnosed and treated for Charcot, and being involved in this study

1. I give permission for a copy of this consent form to be kept confidentially and securely by the Norwich Clinical Trials Unit.
2. I am happy to be contacted to receive updates on how the study

is progressing and to be informed about the results of the study

at the end

1. I agree to take part in the study.

………………………………………. ……………... …………………………………….

Name of the patient (Print) Date Patient’s signature

………………………………………. ……………... ……………………………………….

Name of person taking consent (Print) Date Signature

Original to be retained and filed in the site file. 1 copy to patient, 1 copy to be filed in patient’s notes
