# Supplementary material for: A randomised feasibility study of serial magnetic resonance imaging to reduce treatment times in Charcot neuroarthropathy in people with diabetes (CADOM): a protocol
Source: Pilot Feasibility Stud. 2020 Jun 16;6:85. doi: 10.1186/s40814-020-00611-3 (PMC7296621; doi:10.1186/s40814-020-00611-3)
Supplement: Supplementary file 3 — Additional file 3. Informed consent form—qualitative interviews. [file 40814_2020_611_MOESM3_ESM.docx]

**Interviews**

**Experiences of being treated for Charcot neuroarthropathy and views about taking part in the clinical trial.**

**(Short title: CADOM)**

**Charcot neuroArthropathy Diagnostic Outcome Measures**

**Patient Consent Form**

Principal Investigator: ……………………………………………………..

Patient Study ID: …..……………….. Initials: ………………

Please initial each box

1. I confirm that I have read and understand the information sheet

Version 1.1 dated 25^th^ August 2017 for the above study. I have had the opportunity to ask questions and been given satisfactory answers.

1. I have been given a full explanation of the purpose of the study and

what I will be expected to do.

1. I understand that my medical notes and data collected during the study

YES

NO

may be looked at by individuals from the Clinical Trials Unit at the

University of East Anglia, from regulatory authorities or from the NHS

Trust, where it is relevant to my taking part in this research I give permission for these individuals to have access to my records.

1. I understand that my participation in the interview is voluntary and that

I am free to withdraw from the study at any time, without having to give a reason.

1. I understand that the interview will be recorded on a digital recorder.

I give permission for doing this.

1. I understand that the recordings will be saved on a secure computer at the University of East Anglia. The recordings will be destroyed at the end of the study. The transcripts will be kept for 15 years.
2. I consent to the storage including electronic, of personal information for

the purposes of this study. I understand that any information that could

identify me will be kept strictly confidential and that no personal

information will be included in the study report, my thesis, or other publication.

1. I understand that what I say during the interview is confidential, in

accordance with the Data Protection Act. However, you must be aware

that if you tell the interviewer something which shows that there is a

significant risk to you or someone else, they may need to pass this information on.

If this happens, they will discuss it with you first before anyone else is told

YES

NO

1. I am happy to be contacted to receive updates on how the study is progressing and to be informed about the results of the study

at the end.

1. I give permission for a copy of this consent form to be kept confidentially and securely by the Norwich Clinical Trials Unit.
2. I agree to take part in an interview for the above study.

………………………………………. ……………... …………………………………….

Name of the patient (Print) Date Patient’s signature

………………………………………. ……………... ……………………………………….

Name of person taking consent (Print) Date Signature

Original to be retained and filed in the site file. 1 copy to patient, 1 copy to be filed in patient’s notes
